# Supplementary material for: Investigating the second whitefly population outbreak within a decade in the cotton growing zone of North India
Source: PeerJ. 2024 Jul 4;12:e17476. doi: 10.7717/peerj.17476 (PMC11227808; doi:10.7717/peerj.17476)
Supplement: Supplemental Information 1 [file peerj-12-17476-s001.docx]

**Supplementary Table 1**. Raw data for Fig.-2 i.e comparison of seasonal population dynamics based on experiment under unprotected condition during 2015 and 2022 outbreak

| BG II Cotton Hybrid | | | | |  | N-Bt cotton Hirsutum Variety | | | | |
| --- | --- | --- | --- | --- | --- | --- | --- | --- | --- | --- |
| SMW | 2015 | 2021 | 2022 | ETL |  | SMW | 2015 | 2021 | 2022 | ETL |
| 22 | 0.00 | - | - |  |  | 22 | - | - | - |  |
| 23 | 0.00 | - | - |  |  | 23 | - | - | - |  |
| 24 | 0.00 | 1.20 | 6.50 | 18.00 |  | 24 | - | 0.3 | 11.10 | 18.00 |
| 25 | 0.00 | 1.30 | 7.60 | 18.00 |  | 25 | - | 1.4 | 8.70 | 18.00 |
| 26 | 0.30 | 6.30 | 18.60 | 18.00 |  | 26 | 0.2 | 2.8 | 10.7 | 18.00 |
| 27 | 9.74 | 8.90 | 63.40 | 18.00 |  | 27 | 5.26 | 6.6 | 35.2 | 18.00 |
| 28 | 15.80 | 9.30 | 74.10 | 18.00 |  | 28 | 14.43 | 8.1 | 76.8 | 18.00 |
| 29 | 47.52 | 8.20 | 29.50 | 18.00 |  | 29 | 46.65 | 28.0 | 84.1 | 18.00 |
| 30 | 45.86 | 9.70 | 34.70 | 18.00 |  | 30 | 53.75 | 12.8 | 31.5 | 18.00 |
| 31 | 57.74 | 7.30 | 65.00 | 18.00 |  | 31 | 44.24 | 8.8 | 40.2 | 18.00 |
| 32 | 40.08 | 9.40 | 57.70 | 18.00 |  | 32 | 35.08 | 9.9 | 56.3 | 18.00 |
| 33 | 20.98 | 4.60 | 39.68 | 18.00 |  | 33 | 20.25 | 4.0 | 53.2 | 18.00 |
| 34 | 21.16 | 4.40 | 54.20 | 18.00 |  | 34 | 16.02 | 6.1 | 65 | 18.00 |
| 35 | 14.80 | 9.00 | 16.50 | 18.00 |  | 35 | 13.8 | 12 | 30.1 | 18.00 |
| 36 | 15.20 | 9.90 | 19 | 18.00 |  | 36 | 10.78 | 17.9 | 30.2 | 18.00 |
| 37 | 15.80 | 12.10 | 19.1 | 18.00 |  | 37 | 11.62 | 17 | 21.2 | 18.00 |
| 38 | 16.47 | 13.70 | 11 | 18.00 |  | 38 | 11.53 | 15 | 31.3 | 18.00 |
| 39 | 14.87 | 6.20 | 19.5 | 18.00 |  | 39 | 10.9 | 7.8 | 14.5 | 18.00 |
| 40 | 15.13 | 6.00 | 10.5 | 18.00 |  | 40 | 10.53 | 6.2 | 25 | 18.00 |
| 41 | 14.47 | 2.60 | 1.7 | 18.00 |  | 41 | 9.58 | 3.2 | 8.8 | 18.00 |
| 42 | 13.30 | 3.20 | 0.6 | 18.00 |  | 42 | 7.9 | 2.8 | 3.9 | 18.00 |
| 43 | 12.80 | 2.80 | 0.4 | 18.00 |  | 43 | 7.37 | 2.1 | 0.9 | 18.00 |
| 44 | 7.73 | 1.20 | - | 18.00 |  | 44 | 2.25 | 1.58 | 0.7 | 18.00 |
| 45 | - | - | - | 18.00 |  | 45 | - | - | - | 18.00 |
| 46 | - | - | - | 18.00 |  | 46 | - | - | - | 18.00 |
| Min | 0.30 | 1.20 | 0.40 |  |  | Min | 0.20 | 1.58 | 0.70 |  |
| Max | 57.74 | 13.70 | 74.10 |  |  | Max | 53.75 | 28 | 84.1 |  |

***ETL-Economic Threshold Level**

Supplementary Table 2. Prevailing Weather conditions and average population of White fly/3leaves in the year 2012 at Sirsa

| **SMW** | **MAX**  **Temp**  **°C** | **MIN**  **Temp**  **°C** | **RH**  **(%)**  **M** | **RH**  **(%)**  **E** | **RAIN**  **FALL**  **(mm)** | **Mean**  **Whitefly/3leaves** |
| --- | --- | --- | --- | --- | --- | --- |
|  |  |  |  |  |  |  |
| **24** | 42.5 | 28.9 | 45.3 | 32.4 | 0.0 | 0.0 |
| **25** | 42.3 | 31.2 | 54.0 | 34.0 | 0.0 | 0.0 |
| **26** | 40.5 | 30.3 | 53.4 | 27.6 | 0.0 | 0 |
| **27** | 40.5 | 30.2 | 61.4 | 34.7 | 0.0 | 0.95 |
| **28** | 35.9 | 27.6 | 75.7 | 52.9 | 4.2 | 3.6 |
| **29** | 36.9 | 28.1 | 71.9 | 53.3 | 2.8 | 3.6 |
| **30** | 38.1 | 29.8 | 70.1 | 44.4 | 0.0 | 5.85 |
| **31** | 36.6 | 29.1 | 63.0 | 46.6 | 0.0 | 3.35 |
| **32** | 34.1 | 27.2 | 81.9 | 66.4 | 2.6 | 2.85 |
| **33** | 34.2 | 27.6 | 84.7 | 59.4 | 0.0 | 1.85 |
| **34** | 32.7 | 26.3 | 87.7 | 69.3 | 14.5 | 5.5 |
| **35** | 33.0 | 26.5 | 88.4 | 66.4 | 0.0 | 2.25 |
| **36** | 31.5 | 26.0 | 87.7 | 70.4 | 9.0 | 2.6 |
| **37** | 34.0 | 27.1 | 83.7 | 64.9 | 1.0 | 2.85 |
| **38** | 33.3 | 23.6 | 84.3 | 51.9 | 0.0 | 2.25 |
| **39** | 35.1 | 22.4 | 66.0 | 40.3 | 0.0 | 4.55 |
| **40** | 35.7 | 19.6 | 65.6 | 28.7 | 0.0 | 8.6 |

Supplementary Table 3. Prevailing Weather conditions and average population of White fly/3leaves in the year 2013 at Sirsa

| **SMW** | **MAX**  **Temp**  **°C** | **MIN**  **Temp**  **°C** | **RH**  **(%)**  **M** | **RH**  **(%)**  **E** | **RAIN**  **FALL**  **(mm)** | **Mean**  **Whitefly/3leaves** |
| --- | --- | --- | --- | --- | --- | --- |
| **24** | 34.3 | 25.4 | 74.9 | 72.4 | 4.2 | - |
| **25** | 39.9 | 29.4 | 59.4 | 31.9 | 0.0 | - |
| **26** | 39.9 | 29.5 | 63.0 | 39.7 | 0.0 | 12.9 |
| **27** | 38.0 | 28.6 | 73.0 | 60.4 | 0.3 | 8.8 |
| **28** | 36.9 | 28.6 | 77.0 | 55.7 | 6.6 | 15.9 |
| **29** | 35.9 | 27.9 | 81.0 | 56.0 | 3.1 | 12.4 |
| **30** | 36.1 | 28.1 | 80.7 | 57.1 | 0.0 | 23.0 |
| **31** | 35.9 | 28.3 | 80.7 | 56.4 | 1.3 | 37.0 |
| **32** | 33.1 | 27.2 | 85.4 | 65.6 | 3.3 | 16.5 |
| **33** | 31.5 | 26.0 | 90.0 | 81.3 | 18.8 | 8.1 |
| **34** | 34.4 | 27.1 | 80.6 | 64.6 | 0.9 | 8.9 |
| **35** | 35.4 | 27.3 | 72.9 | 55.6 | 0.0 | 4.1 |
| **36** | 35.2 | 26.0 | 70.0 | 48.9 | 0.0 | 2.1 |
| **37** | 35.9 | 25.8 | 73.0 | 47.1 | 0.0 | 1.4 |
| **38** | 34.3 | 23.8 | 81.6 | 54.3 | 0.3 | 4.9 |
| **39** | 33.6 | 25.4 | 82.6 | 61.0 | 1.0 | 10.2 |
| **40** | 32.7 | 24.2 | 83.3 | 54.1 | 0.0 | 12.6 |
| **41** | 32.7 | 24.3 | 83.6 | 51.1 | 0.0 | 9.8 |
| **42** | 34.2 | 19.8 | 75.6 | 32.0 | 0.0 | 5.0 |

Supplementary Table 4. Prevailing Weather conditions and average population of White fly/3leaves in the year 2014 at Sirsa

| **SMW** | **MAX**  **Temp**  **°C** | **MIN**  **Temp**  **°C** | **RH**  **(%)**  **M** | **RH**  **(%)**  **E** | RAIN  FALL  (mm) | **Mean**  **Whitefly/3leaves** |
| --- | --- | --- | --- | --- | --- | --- |
|  |  |  |  |  |  |  |
| **24** | 41.7 | 27.7 | 54.0 | 45.3 | 0.0 | 0.45 |
| **25** | 40.4 | 28.9 | 53.6 | 36.6 | 0.0 | 0.85 |
| **26** | 37.0 | 27.4 | 65.6 | 55.9 | 1.2 | 3.40 |
| **27** | 36.7 | 27.9 | 73.0 | 56.9 | 11.7 | 5.70 |
| **28** | 40.5 | 29.5 | 56.6 | 41.1 | 0.0 | 9.90 |
| **29** | 36.9 | 28.1 | 76.1 | 59.7 | 0.0 | 29.30 |
| **30** | 28.7 | 27.0 | 80.0 | 62.7 | 4.3 | 19.80 |
| **31** | 35.6 | 27.1 | 90.7 | 66.7 | 0.7 | 9.80 |
| **32** | 35.9 | 28.2 | 79.6 | 60.6 | 0.0 | 9.35 |
| **33** | 36.0 | 27.5 | 73.1 | 56.1 | 0.0 | 13.80 |
| **34** | 32.2 | 28.2 | 84.0 | 53.7 | 0.0 | 15.95 |
| **35** | 33.2 | 26.7 | 82.7 | 73.4 | 3.6 | 17.45 |
| **36** | 31.6 | 25.3 | 90.0 | 75.9 | 12.0 | 25.90 |
| **37** | 33.7 | 24.1 | 87.9 | 68.1 | 8.8 | 35.95 |
| **38** | 34.1 | 24.2 | 75.7 | 53.0 | 0.0 | 23.90 |
| **39** | 34.9 | 23.6 | 64.7 | 46.3 | 0.0 | 15.50 |
| **40** | 35.7 | 22.9 | 68.3 | 52.7 | 0.0 | 9.40 |
| **41** | 32.4 | 18.7 | 79.1 | 49.4 | 0.0 | 8.70 |
| **42** | 30.8 | 15.4 | 73.6 | 42.9 | 0.0 | 5.50 |

Supplementary Table 5. Prevailing Weather conditions and average population of White fly/3leaves in the year 2015 at Sirsa

| **SMW** | **MAX**  **Temp**  **°C** | **MIN**  **Temp**  **°C** | **RH**  **(%)**  **M** | **RH**  **(%)**  **E** | **RAIN**  **FALL**  **(mm)** | **Mean**  **Whitefly/3leaves** |
| --- | --- | --- | --- | --- | --- | --- |
| **24** | 38.2 | 26.5 | 67.9 | 44.6 | 1.1 |  |
| **25** | 39.3 | 27.8 | 66.9 | 39.1 | 1.2 |  |
| **26** | 38.9 | 27.0 | 78.9 | 45.7 | 0.9 | 0.00 |
| **27** | 36.2 | 26.9 | 76.4 | 56.0 | 6.9 | 4.90 |
| **28** | 32.6 | 26.2 | 86.7 | 66.0 | 3.3 | 14.10 |
| **29** | 36.4 | 27.7 | 71.3 | 56.1 | 0.0 | 50.00 |
| **30** | 33.8 | 26.0 | 82.3 | 65.4 | 2.3 | 45.80 |
| **31** | 31.8 | 25.5 | 92.0 | 76.0 | 1.9 | 46.92 |
| **32** | 34.8 | 27.7 | 86.1 | 65.4 | 2.1 | 33.76 |
| **33** | 34.1 | 26.7 | 85.9 | 65.6 | 2.9 | 21.74 |
| **34** | 35.1 | 26.9 | 76.4 | 57.1 | 0.0 | 17.04 |
| **35** | 36.3 | 27.3 | 76.0 | 50.9 | 0.0 | 9.90 |
| **36** | 36.2 | 24.3 | 66.9 | 46.0 | 0.0 | 10.07 |
| **37** | 37.6 | 24.3 | 76.4 | 46.1 | 0.0 | 11.20 |
| **38** | 33.4 | 23.3 | 86.3 | 62.6 | 2.5 | 11.40 |
| **39** | 34.3 | 21.7 | 80.0 | 50.7 | 0.0 | 10.27 |
| **40** | 36.0 | 20.6 | 74.7 | 38.3 | 0.0 | 10.40 |
| **41** | 35.7 | 20.9 | 67.4 | 32.0 | 0.0 | 9.20 |
| **42** | 35.3 | 21.3 | 81.4 | 37.4 | 0.0 | 7.63 |

Supplementary Table 6. Prevailing Weather conditions and average population of White fly/3leaves in the year 2016 at Sirsa

| **SMW** | **MAX**  **Temp**  **°C** | **MIN**  **Temp**  **°C** | **RH**  **(%)**  **M** | **RH**  **(%)**  **E** | **RAIN**  **FALL**  **(mm)** | **Mean**  **Whitefly/3leaves** |
| --- | --- | --- | --- | --- | --- | --- |
|  |  |  |  |  |  |  |
| **24** | 39.9 | 27.5 | 66.4 | 44.6 | 1.1 | 8.60 |
| **25** | 40.1 | 28.9 | 78.7 | 35.3 | 0.0 | 10.15 |
| **26** | 39.7 | 30.3 | 76.7 | 49.0 | 0.0 | 10.00 |
| **27** | 34.6 | 26.8 | 82.7 | 67.9 | 19.0 | 14.24 |
| **28** | 36.5 | 27.7 | 78.3 | 58.7 | 0.0 | 14.25 |
| **29** | 36.3 | 27.5 | 80.7 | 57.7 | 0.6 | 12.10 |
| **30** | 35.8 | 28.0 | 79.9 | 59.7 | 0.0 | 17.45 |
| **31** | 35.5 | 27.4 | 81.9 | 62.1 | 0.0 | 15.70 |
| **32** | 33.9 | 26.9 | 87.1 | 67.4 | 0.0 | 25.35 |
| **33** | 35.4 | 27.1 | 79.1 | 61.9 | 2.8 | 21.85 |
| **34** | 33.2 | 26.6 | 85.3 | 69.1 | 5.2 | 15.50 |
| **35** | 31.9 | 25.3 | 91.4 | 73.9 | 4.2 | 11.55 |
| **36** | 34.6 | 25.5 | 79.1 | 55.3 | 0.0 | 9.55 |
| **37** | 35.1 | 25.1 | 78.6 | 53.1 | 0.0 | 10.00 |
| **38** | 35.3 | 25.3 | 80.6 | 55.7 | 0.0 | 5.90 |
| **39** | 35.0 | 24.9 | 82.4 | 54.3 | 0.0 | 15.40 |
| **40** | 35.1 | 25.5 | 85.6 | 59.1 | 0.0 | 5.40 |
| **41** | 35.1 | 20.7 | 72.1 | 39.6 | 0.0 | 3.90 |
| **42** | 35.4 | 16.0 | 67.4 | 35.1 | 0.0 | 2.00 |

Supplementary Table 7. Prevailing Weather conditions and average population of White fly/3leaves in the year 2017 at Sirsa

| **SMW** | **MAX**  **Temp**  **°C** | **MIN**  **Temp**  **°C** | **RH**  **(%)**  **M** | **RH**  **(%)**  **E** | **RAIN**  **FALL**  **(mm)** | **Mean**  **Whitefly/3leaves** |
| --- | --- | --- | --- | --- | --- | --- |
|  |  |  |  |  |  |  |
| **24** | 38.7 | 25.2 | 59.6 | 39.4 | 1.8 | 0.43 |
| **25** | 35.6 | 25.2 | 79.3 | 50.6 | 5.5 | 1.34 |
| **26** | 34.3 | 27.4 | 80.0 | 65.3 | 0.0 | 9.88 |
| **27** | 38.3 | 28.4 | 74.7 | 48.9 | 0.0 | 12.76 |
| **28** | 38.1 | 28.9 | 70.3 | 49.0 | 0.0 | 15.91 |
| **29** | 36.2 | 27.3 | 82.4 | 58.1 | 4.0 | 38.70 |
| **30** | 35.2 | 27.4 | 84.3 | 62.4 | 0.0 | 40.75 |
| **31** | 34.9 | 26.6 | 81.9 | 60.7 | 0.4 | 18.10 |
| **32** | 37.7 | 26.7 | 76.4 | 53.1 | 2.0 | 28.75 |
| **33** | 37.3 | 26.7 | 78.4 | 49.9 | 0.0 | 21.60 |
| **34** | 35.9 | 26.0 | 81.1 | 61.1 | 0.2 | 10.25 |
| **35** | 32.8 | 25.6 | 83.3 | 71.3 | 15.4 | 26.64 |
| **36** | 34.8 | 24.9 | 87.3 | 62.4 | 2.3 | 20.70 |
| **37** | 34.8 | 25.7 | 84.7 | 60.4 | 0.0 | 15.00 |
| **38** | 35.2 | 23.0 | 77.6 | 47.9 | 0.0 | 7.80 |
| **39** | 36.7 | 22.7 | 72.1 | 40.7 | 0.0 | 11.75 |
| **40** | 36.8 | 19.7 | 66.4 | 33.7 | 0.0 | 7.45 |
| **41** | 35.8 | 20.0 | 84.1 | 35.1 | 0.0 | 6.75 |
| **42** | 36.0 | 16.5 | 76.6 | 21.3 | 0.0 | 5.85 |

Supplementary Table 8. Prevailing Weather conditions and average population of White fly/3leaves in the year 2018 at Sirsa

| **SMW** | **MAX**  **Temp**  **°C** | **MIN**  **Temp**  **°C** | **RH**  **(%)**  **M** | **RH**  **(%)**  **E** | **RAIN**  **FALL**  **(mm)** | **Mean**  **Whitefly/3leaves** |
| --- | --- | --- | --- | --- | --- | --- |
|  |  |  |  |  |  |  |
| **24** | 39.6 | 24.8 | 62.9 | 45.9 | 0.0 | 6.00 |
| **25** | 38.6 | 21.8 | 76.1 | 49.0 | 1.1 | 10.80 |
| **26** | 35.6 | 21.4 | 76.9 | 63.3 | 8.8 | 14.70 |
| **27** | 36.0 | 24.4 | 82.0 | 62.1 | 2.9 | 25.40 |
| **28** | 38.0 | 27.0 | 72.4 | 51.4 | 0.0 | 28.10 |
| **29** | 35.3 | 26.5 | 77.3 | 64.9 | 1.3 | 26.20 |
| **30** | 35.1 | 26.4 | 84.4 | 66.4 | 4.8 | 27.30 |
| **31** | 36.3 | 27.4 | 66.9 | 53.7 | 0.0 | 18.70 |
| **32** | 34.1 | 25.8 | 78.6 | 64.3 | 3.7 | 15.50 |
| **33** | 35.5 | 26.1 | 80.4 | 59.9 | 0.3 | 22.90 |
| **34** | 35.8 | 27.4 | 81.7 | 60.3 | 0.0 | 11.90 |
| **35** | 35.6 | 27.0 | 74.4 | 54.9 | 0.0 | 9.70 |
| **36** | 35.1 | 26.6 | 74.1 | 58.7 | 0.0 | 14.40 |
| **37** | 35.1 | 25.4 | 77.1 | 54.0 | 0.8 | 9.90 |
| **38** | 33.7 | 24.1 | 73.7 | 55.1 | 5.3 | 11.00 |
| **39** | 32.0 | 22.4 | 89.3 | 64.3 | 3.0 | 14.10 |
| **40** | 34.4 | 22.0 | 89.6 | 45.4 | 0.0 | 9.90 |
| **41** | 32.3 | 19.2 | 75.1 | 48.4 | 0.0 | 8.60 |
| **42** | 33.4 | 18.5 | 71.1 | 29.4 | 0.0 | 5.80 |

Supplementary Table 9. Prevailing Weather conditions and average population of White fly/3leaves in the year 2019 at Sirsa

| **SMW** | **MAX**  **Temp**  **°C** | **MIN**  **Temp**  **°C** | **RH**  **(%)**  **M** | **RH**  **(%)**  **E** | **RAIN**  **FALL**  **(mm)** | **Mean**  **Whitefly/3leaves** |
| --- | --- | --- | --- | --- | --- | --- |
|  |  |  |  |  |  |  |
| **24** | 42.2 | 29.6 | 53.9 | 30.7 | 7.5 | 4.40 |
| **25** | 37.6 | 26.0 | 70.1 | 46.1 | 2.0 | 3.55 |
| **26** | 39.9 | 26.9 | 62.3 | 41.7 | 0.0 | 12.40 |
| **27** | 38.3 | 27.1 | 63.6 | 47.3 | 1.8 | 14.60 |
| **28** | 36.1 | 26.2 | 71.7 | 56.3 | 1.2 | 14.45 |
| **29** | 33.3 | 22.0 | 83.1 | 66.0 | 10.5 | 13.25 |
| **30** | 33.3 | 22.1 | 83.6 | 71.6 | 12.4 | 26.45 |
| **31** | 34.8 | 22.9 | 80.9 | 65.9 | 0.0 | 14.70 |
| **32** | 36.9 | 24.7 | 72.0 | 56.7 | 0.7 | 15.70 |
| **33** | 34.0 | 21.9 | 79.3 | 67.4 | 5.6 | 17.05 |
| **34** | 35.9 | 27.2 | 73.1 | 59.1 | 0.0 | 10.95 |
| **35** | 36.3 | 27.4 | 76.6 | 59.9 | 0.0 | 7.95 |
| **36** | 36.1 | 27.0 | 80.0 | 59.1 | 0.0 | 11.50 |
| **37** | 36.8 | 27.4 | 77.7 | 56.0 | 0.0 | 17.25 |
| **38** | 34.5 | 25.1 | 78.3 | 58.3 | 0.2 | 17.25 |
| **39** | 33.5 | 22.2 | 75.7 | 59.0 | 0.0 | 13.15 |
| **40** | 31.7 | 21.0 | 84.0 | 58.0 | 1.9 | 14.40 |
| **41** | 33.1 | 19.6 | 75.9 | 43.9 | 0.0 | 14.15 |
| **42** | 32.3 | 19.9 | 73.6 | 42.6 | 0.2 | 10.75 |

Supplementary Table 10. Prevailing Weather conditions and average population of White fly/3leaves in the year 2020 at Sirsa

| **SMW** | **MAX**  **Temp**  **°C** | **MIN**  **Temp**  **°C** | **RH**  **(%)**  **M** | **RH**  **(%)**  **E** | **RAIN**  **FALL**  **(mm)** | **Mean**  **Whitefly/3leaves** |
| --- | --- | --- | --- | --- | --- | --- |
|  |  |  |  |  |  |  |
| **24** | 42.9 | 30.1 | 56.1 | 63.1 | 0.0 | 0.45 |
| **25** | 40.6 | 29.9 | 61.0 | 40.0 | 0.0 | 3.35 |
| **26** | 38.6 | 29.8 | 73.9 | 47.7 | 1.5 | 5.70 |
| **27** | 38.8 | 29.3 | 74.6 | 48.3 | 3.1 | 18.50 |
| **28** | 37.0 | 26.7 | 78.9 | 51.6 | 6.2 | 9.05 |
| **29** | 34.8 | 26.2 | 87.3 | 61.1 | 12.8 | 18.80 |
| **30** | 36.4 | 27.0 | 74.9 | 55.6 | 0.0 | 10.95 |
| **31** | 36.5 | 26.9 | 72.1 | 60.1 | 0.0 | 12.15 |
| **32** | 36.5 | 26.8 | 78.4 | 60.3 | 2.3 | 12.35 |
| **33** | 36.1 | 26.5 | 84.1 | 60.4 | 0.3 | 10.35 |
| **34** | 33.4 | 26.1 | 85.3 | 73.1 | 2.6 | 6.05 |
| **35** | 33.9 | 25.8 | 86.3 | 61.9 | 0.3 | 16.85 |
| **36** | 34.8 | 25.7 | 87.7 | 61.0 | 5.8 | 42.25 |
| **37** | 37.0 | 27.1 | 80.0 | 51.7 | 0.0 | 27.90 |
| **38** | 36.6 | 26.9 | 71.7 | 54.4 | 0.0 | 45.60 |
| **39** | 36.3 | 25.8 | 67.1 | 42.9 | 0.0 | 34.90 |
| **40** | 36.2 | 25.1 | 83.7 | 50.7 | 0.0 | 10.90 |
| **41** | 35.6 | 23.0 | 66.1 | 30.3 | 0.0 | 9.55 |
| **42** | 34.0 | 18.4 | 59.1 | 25.7 | 0.0 | 6.30 |

Supplementary Table 11. Prevailing Weather conditions and average population of White fly/3leaves in the year 2021 at Sirsa

| **SMW** | **MAX**  **Temp**  **°C** | **MIN**  **Temp**  **°C** | **RH**  **(%)**  **M** | **RH**  **(%)**  **E** | **RAIN**  **FALL**  **(mm)** | **Mean**  **Whitefly/3leaves** |
| --- | --- | --- | --- | --- | --- | --- |
|  |  |  |  |  |  |  |
| **24** | 37.4 | 27.8 | 68.7 | 44.1 | 18.24 | 3.03 |
| **25** | 39.1 | 28.6 | 66.9 | 41.3 | 1.37 | 4.48 |
| **26** | 41.7 | 29.7 | 57.6 | 31.9 | 0.00 | 6.73 |
| **27** | 40.3 | 30.5 | 58.4 | 37.3 | 0.00 | 12.29 |
| **28** | 37.9 | 28.5 | 68.6 | 51.4 | 0.50 | 12.75 |
| **29** | 37.4 | 27.1 | 79 | 55.6 | 0.26 | 25.86 |
| **30** | 33.8 | 27.1 | 85.3 | 73.1 | 6.64 | 18.95 |
| **31** | 34.5 | 26.7 | 87.4 | 67.4 | 0.21 | 14.25 |
| **32** | 37.1 | 27.6 | 70.4 | 52 | 0.00 | 14.12 |
| **33** | 38.4 | 28.9 | 66.4 | 48.3 | 0.37 | 11.05 |
| **34** | 35.1 | 26.3 | 82.7 | 62.4 | 4.36 | 9.46 |
| **35** | 34.6 | 25.6 | 83 | 60.9 | 0.00 | 13.11 |
| **36** | 33.1 | 24.9 | 90.9 | 70.6 | 2.81 | 20.22 |
| **37** | 32.3 | 23.9 | 81.9 | 67.6 | 5.87 | 18.41 |
| **38** | 32.6 | 24.5 | 79.4 | 67.7 | 4.67 | 19.37 |
| **39** | 33.5 | 23.8 | 86.9 | 65.6 | 0.00 | 14.05 |
| **40** | 34.6 | 24.5 | 85.4 | 57 | 0.57 | 10.22 |
| **41** | 35.7 | 22.2 | 71.9 | 36.1 | 0.00 | 8.23 |
| **42** | 32.9 | 20.2 | 64.1 | 38.3 | 0.00 | 6.89 |

Supplementary Table 12. Prevailing Weather conditions and average population of White fly/3leaves in the year 2022 at Sirsa

| **SMW** | **MAX**  **Temp**  **°C** | **MIN**  **Temp**  **°C** | **RH**  **(%)**  **M** | **RH**  **(%)**  **E** | **RAIN**  **FALL**  **(mm)** | **Mean**  **Whitefly/3leaves** |
| --- | --- | --- | --- | --- | --- | --- |
|  |  |  |  |  |  |  |
| **24** | 43.20 | 28.30 | 54.57 | 36.86 | 0.00 | 10.70 |
| **25** | 33.10 | 24.50 | 84.43 | 56.14 | 52.40 | 8.65 |
| **26** | 39.80 | 27.00 | 57.29 | 43.00 | 5.80 | 10.35 |
| **27** | 36.50 | 28.14 | 71.86 | 55.29 | 1.20 | 34.16 |
| **28** | 35.60 | 26.43 | 77.14 | 71.00 | 77.50 | 77.10 |
| **29** | 35.20 | 26.29 | 83.00 | 77.29 | 78.80 | 84.20 |
| **30** | 33.00 | 26.29 | 90.29 | 76.71 | 94.20 | 32.35 |
| **31** | 32.30 | 26.21 | 89.00 | 80.71 | 67.40 | 38.75 |
| **32** | 33.70 | 26.71 | 86.71 | 67.57 | 0.00 | 59.60 |
| **33** | 33.50 | 25.79 | 86.57 | 68.43 | 0.00 | 40.35 |
| **34** | 34.30 | 26.40 | 89.14 | 62.29 | 0.00 | 60.15 |
| **35** | 35.50 | 25.79 | 85.71 | 58.29 | 6.80 | 31.20 |
| **36** | 36.70 | 25.97 | 86.14 | 55.00 | 0.00 | 27.55 |
| **37** | 35.30 | 25.71 | 84.00 | 62.00 | 0.00 | 19.80 |
| **38** | 34.60 | 24.57 | 87.57 | 68.00 | 16.80 | 24.90 |
| **39** | 30.30 | 23.04 | 83.14 | 66.14 | 20.20 | 14.85 |
| **40** | 33.50 | 22.04 | 79.14 | 48.57 | 0.00 | 20.15 |
| **41** | 27.00 | 19.51 | 86.86 | 64.14 | 1.00 | 9.25 |
| **42** | 32.90 | 17.66 | 75.29 | 37.43 | 0.00 | 2.50 |

Supplementary Table 13: **Year wise regression equation between** Prevailing Weather conditions and average population of White fly/3leaves at Sirsa

| Year | Regression Equation | R^2^ |
| --- | --- | --- |
| 2012 | Y= -0.22*Tmax-0.30*Tmin+0.05*RH (M)-0.10*RH (E)+0.17*Rainfall+19.98 | 0.51 |
| 2013 | Y= 2.36*Tmax+1.87*Tmin+1.69*RH (M)-0.33*RH (E)-0.29*Rainfall-234.22 | 0.64 |
| 2014 | Y= -0.49*Tmax+0.22*Tmin+0.22*RH (M)+0.25*RH (E)+0.13*Rainfall-4.94 | 0.42 |
| 2015 | Y= -1.21*Tmax-0.50*Tmin-0.77*RH (M)+1.42*RH (E)-3.42*Rainfall+61.67 | 0.62 |
| 2016 | Y= -1.40*Tmax+1.39*Tmin-0.31*RH (M)+0.17*RH (E)-0.12*Rainfall+41.00 | 0.53 |
| 2017 | Y= 5.51*Tmax-0.78*Tmin+0.98*RH (M)+0.62*RH (E)+0.65*Rainfall-272.47 | 0.47 |
| 2018 | Y= 0.69*Tmax+0.67*Tmin+0.19*RH (M)+0.31*RH (E)+0.09*Rainfall-56.97 | 0.44 |
| 2019 | Y= 0.25*Tmax-0.70*Tmin-0.14*RH (M)+0.36*RH (E)+0.10*Rainfall+11.89 | 0.47 |
| 2020 | Y= -3.26*Tmax+2.17*Tmin+0.06*RH (M)-0.21*RH (E)+0.07*Rainfall+83.99 | 0.14 |
| 2021 | Y= 4.59*Tmax-3.51*Tmin-0.54*RH (M)+1.25*RH (E)-0.15*Rainfall-85.85 | 0.65 |
| 2022 | Y= 4.61*Tmax-1.46*Tmin+0.10*RH (M)+1.86*RH (E)-0.05*Rainfall-210.58 | 0.56 |
